# Magnetization switching of multi-state magnetic structures with current-induced torques

**DOI:** 10.1038/s41598-018-33554-0

**Published:** 2018-10-11

**Authors:** Shubhankar Das, Liran Avraham, Yevgeniy Telepinsky, Vladislav Mor, Moty Schultz, Lior Klein

**Affiliations:** Department of Physics, Nano-magnetism Research Center, Institute of Nanotechnology and Advanced Materials, Bar-Ilan University, Ramat-Gan, 52900 Israel

## Abstract

Spintronic devices often require the ability to locally change the magnetic configuration of ferromagnetic structures on a sub-micron scale. A promising route for achieving this goal is the use of heavy metal/ferromagnetic heterostructures where current flowing through the heavy metal layer generates field-like and anti-damping like torques on the magnetic layer. Commonly, such torques are used to switch magnets with a uniaxial anisotropy between two uniformly magnetized states. Here, we use such torques to switch magnetization in Ta/Ni_0.80_Fe_0.20_ heterostructures with uniaxial and biaxial anisotropy, where in the latter the magnetization is non-uniform. The anisotropies are induced by shape and the magnetic state is monitored using the planar Hall effect. As structures with several easy axes induced by shape can be part of a magnetic memory element, the results pave the way for multi-level magnetic memory with spin-orbit torque switching.

## Introduction

A common requirement for spintronic devices is an efficient and scalable method to manipulate the magnetic configuration of a ferromagnetic (FM) structure on a sub-micron scale. While spin transfer torques (STT) induced by injecting a spin polarized current into a FM-layer provide a satisfactory solution in many respects^1–7^, it is increasingly realized that charge current injection used for STT is potentially detrimental, e.g., for magnetic tunnel junctions (MTJs). Consequently, there is an increasing interest shifting from the use of charge currents to spin currents which are efficiently generated in heavy metal/ferromagnetic (HM/FM) heterostructure^8–15^. A flowing charge current in HM/FM heterostructures generates spin-orbit torques (SOTs), owing to the spin Hall effect (SHE) and Rashba effect at the interface, which affect the magnetic layer without injecting a charge current into the FM layer^16–22^. Furthermore, those effects create two types of torques on the magnetic layer known as field like and an anti-damping like torques. It has been experimentally demonstrated that SOTs effectively switch the magnetization in uniaxial anisotropy HM/FM heterostructures between two uniformly magnetized states^23–36^, with the assistance of an external in-plane magnetic field, although there are attempts to overcome this requirement by special engineering of the heterostructure^30^.

Here we study the SOTs induced magnetization switching of multi-state magnetic structures which consist of Ta/Ni_0.8_Fe_0.2_/Ti heterostructures with in-plane magnetization. The magnetic anisotropy induced by shape is either uniaxial or biaxial achieved by elongated ellipses or two crossing ellipses, respectively. Furthermore, the two crossing ellipses exhibit effective bi-axial anisotropy only in the crossing area; thus, the remanent magnetization direction is non-uniform. We monitor the magnetic configuration while generating the SOTs by using the planar Hall effect (PHE) and we demonstrate the important contribution of the anti-damping torque. In addition, we show field-free current-induced switching in the two types of structures, providing a pathway for practical design of spintronic devices based on bi and multi-easy axes in-plane magnetized heterostructures. In particular, as such structures may be one of the magnetic electrodes in MTJs, these results indicate the feasibility of multi-state magnetic memory with SOTs used for write operations.

## Results

### The studied devices

**Figure 1:**
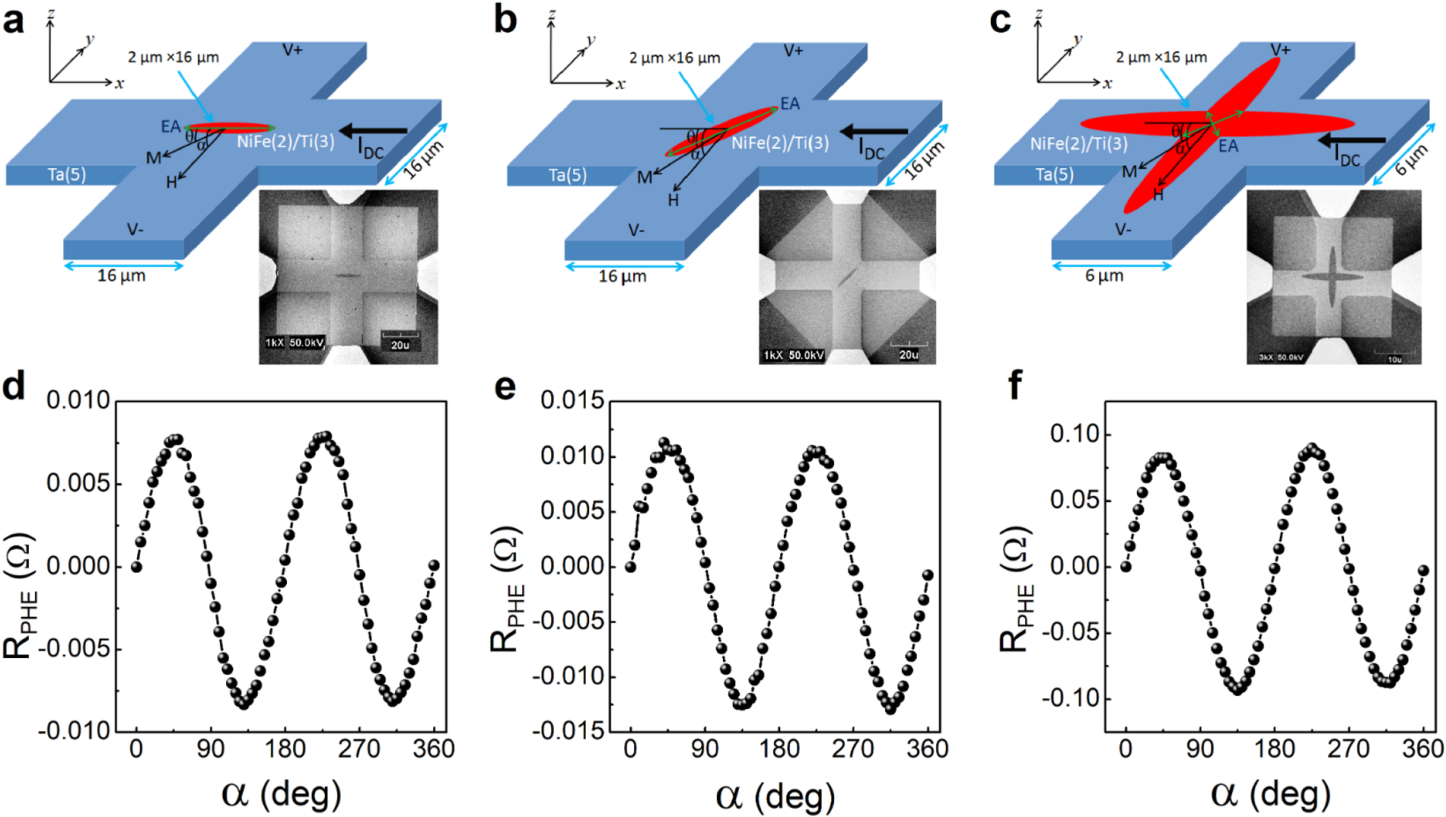
The structure and characterization of the devices. (**a**,**b** and **c**) Schematic illustrations and scanning electron microscopy images of Devices 1, 2 and 3, respectively. The angle *α* is between the current **I** and the applied field **H** and *θ* is between **I** and the magnetization **m** in all structures. The structures consist of Ta(5 nm), Ni_0.80_Fe_0.20_ (2 nm) and Ti(3 nm). (**d**,**e** and **f**) R_PHE_ is measured with a field of 100 Oe as a function of *α* of Devices 1, 2 and 3, respectively.

Our devices are magnetic structures made of Ni_0.8_Fe_0.2_ (NiFe) on top of a heavy metal (*β*-Ta) cross-like structure (see Fig. 1). The heterostructure consists of *β*-Ta(5)/NiFe(2)/Ti(3) with the numbers in the parenthesis indicating the layer thickness in nanometer. The *β*-Ta layer acts as a source of SOT by flowing an in-plane current in the structure, whereas Ti is a capping layer. We note that the Ti-layer is efficiently thinner due to oxidation and has insignificant spin Hall angle; therefore, its contribution to SOT is negligible.

Three types of devices are used: a) Device 1 (Fig. 1(a)): the magnetic structure is in the shape of an ellipse with its principal axis parallel to the cross arms, b) Device 2 (Fig. 1(b)): the magnetic structure is in the shape of an ellipse with its principal axis at 45 degrees with the cross arms and c) Device 3 (Fig. 1(c)): the magnetic structure is in the shape of two crossing ellipses with their principal axes parallel to the cross arms. As we show below, the single ellipse structures effectively behave as single magnetic domains with uniaxial anisotropy parallel to the long axis of the ellipse, and in the double-ellipse structures the overlap area of the ellipses has an effective bi-axial anisotropy with the easy axes at 45 degrees with respect to the arms of the Ta cross. In both types of structures, the magnetic anisotropy is shape-induced; namely, it is dominated by magnetostatic energy considerations. We use PHE measurements for magnetic characterization of the NiFe structures, and we drive current through the arms of the Ta cross to induce SOTs.

Figure 1(d,e and f) show PHE measurements of the NiFe structures in the three devices, respectively. We plot planar Hall resistance (R_PHE_), which is the voltage across one arm of the Ta cross, divided by the current driven through the other arm. In magnetic conductors for which crystal symmetries are averaged out, R_PHE_ is given by

1 $${{\rm{R}}}_{{\rm{PHE}}}=\frac{1}{2}{\rm{\Delta }}{\rm{R}}\,{\rm{s}}{\rm{i}}{\rm{n}}2\theta $$

where ΔR is the anisotropic magnetoresistance amplitude and *θ* is the angle between the magnetization and current direction. The PHE measurements of our devices, performed with an in-plane field of 100 Oe, are consistent with equation (1) indicating that R_PHE_ reflects the magnetic state of the NiFe structures.

**Figure 2:**
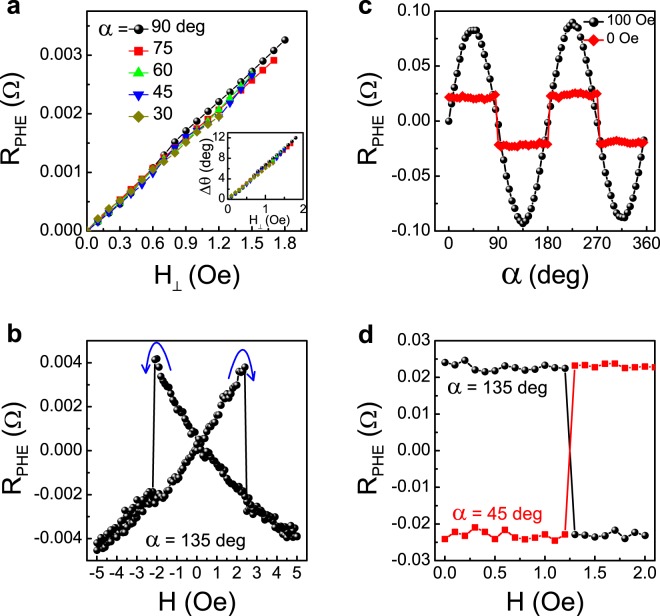
Magnetic characterization of Devices 1 and 3. Device 1: (**a**) R_PHE_ as a function of the field component perpendicular to the long axis of the ellipse for fields applied at different angles. Inset: the corresponding change in the magnetization direction vs H_⊥_ for various field direction. (**b**) R_PHE_ vs field at *α* = 135°. Device 3: (**c**) R_PHE_ as a function of *α* at 100 Oe and after the field is switched off. (**d**) Switching between two states with a field applied along 135° (circle) and 45° (square). The data are taken with the field switched off.

The effective uniaxial single magnetic domain behavior of Device 1 is demonstrated in Fig. 2(a). The structure is fully magnetized with a field parallel to the long axis of the ellipse. Subsequently, R_PHE_ is measured with applied fields at different angles and a scaling with the perpendicular component of the field (H_⊥_) is demonstrated. The corresponding change in the magnetization direction (Δ*θ*), calculated from equation (1), as a function of H_⊥_ for various field angles is shown as inset of Fig. 2(a). For H_⊥_ ≪ H_*k*_, we expect Δ*θ* ~ H_⊥_/H_K_^37^, thus the anisotropy field is calculated from the slope of Δ*θ* vs H_⊥_ curve, which yields 8.4 Oe. Furthermore, it is found that the magnetic hysteresis of the structure exhibits very sharp switching behavior similar to the behavior of single magnetic domains (Fig. 2(b))^37^.

The effective bi-axial magnetic anisotropy of Device 3 is demonstrated in Fig. 2(c). The structure is fully magnetized with an external field of 100 Oe at different angles and R_PHE_ is measured with the field on and after the field is set to zero. We see two plateaus in the remanent state which are indicative of bi-axial magnetic anisotropy with the easy axes at 45 degrees relative to the long axes of the ellipses. We note that the absolute value of R_PHE_ in the plateaus is smaller than the maximum absolute value of R_PHE_ with the field on. The reason for that is that when a 100 Oe field is applied, the magnetization in the entire permalloy structure points in the field direction. On the other hand, in the remanent states, only the magnetization in the overlap area is along one of the two easy axes whereas the magnetization in the arms is at angles of 0, 90, 180 or 270 degrees with respect to the current, and for these angles R_PHE_ is zero. Since R_PHE_ reflects the magnetization in the overlap area and also in parts of the arms, the absolute value of R_PHE_ in the remanent state is lower. Furthermore, Fig. 2(d) shows very sharp switchings between the different remanent states. Please note that a comprehensive study of the magnetic properties of structures such as Device 3 is presented in refs^38,39^. Additional magnetic characterization is presented in the supplementary material.

### Current-induced Torques

Here we study changes in the magnetic orientation of the NiFe structures in Devices 1 and 2 as a result of driving currents through the arms of the Ta cross. In the limit of small currents, driving a current through one arm and measuring the induced transverse voltage across the other arm yields just the usual PHE effect. However, as the driving current is increased, the current, in addition to being a probing current, also affects the magnetization state. To determine the contribution associated with the induced change in the magnetic orientation we define ΔV as the sum of the transverse voltages for positive and negative current biases. When the current does not induce any change in the magnetization, ΔV is expected to be zero. However, when the current itself changes the magnetization and opposite currents have opposite effects on the magnetic orientation, ΔV is not zero and its magnitude can be used to determine the current-induced changes in the magnetic orientation. We note that if the current induced changes in R_PHE_ are approximately linear, then we expect ΔV which is proportional to changes in R_PHE_ to be quadratic in the current.

**Figure 3:**
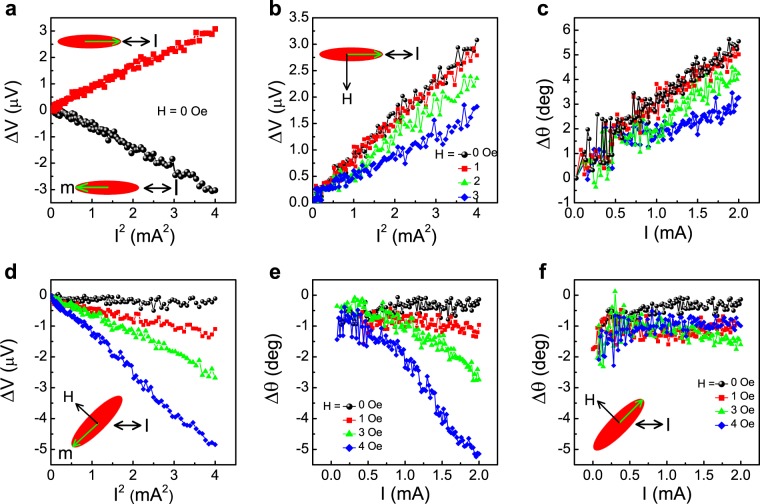
Current-induced magnetic reorientation in Devices 1 and 2. Device 1: (**a**) ΔV vs I^2^ with current parallel to the long axis of the ellipse for opposite initial magnetic states. (**b** and **c**) ΔV vs I^2^ and the corresponding change in the angle of magnetization as a function of current, respectively, for various initial magnetic states. Device 2: (**d**) ΔV vs I^2^ for current along the horizontal Ta-arm for different initial magnetic states. The corresponding change in *θ* for the magnetization rotation due to current is shown in (**e**). (**f**) Δ*θ* vs I for various initial magnetic states when the applied field increases the angle between **I** and **m**. The schematics of the configurations are shown in the insets.

Figure 3(a) shows ΔV vs. I^2^ for two opposite remanent states with the current flowing along the easy axis of the ellipse in Device 1. We note that reversing the magnetization reverses the sign of the slope which is a direct indication that the induced ΔV is related to intrinsic changes in the magnetization. Namely, in these measurements the current induces a change in the magnetic orientation and ΔV reflects the degree of this change.

We now turn to probe the effect of the current for other initial states of the magnetization which are set by applying a field of 1, 2 and 3 Oe along the hard axis which yield magnetic rotation of 6.7, 13 and 18.8 degrees, respectively. Figure 3(b) shows ΔV vs. I^2^ for the different initial states. Dividing ΔV by the current yields the change in R_PHE_ from which (by using equation (1)) Δ*θ* is calculated and Fig. 3(c) shows the extracted change in the magnetic orientation as a function of current. We note a decrease in the slope as the magnetization is rotated away from the current direction.

The same trend is also observed in Device 2 for which the angle between the current and remanent magnetization is 45 deg. When the applied field along the hard axis decreases this angle, the slope of ΔV vs. I^2^ increases (Fig. 3(d and e)) and when the applied field along the hard axis further increases this angle the slope remains very small (Fig. 3(f)). Note that the slope is also affected by the actual anisotropy field of the devices and whereas the anisotropy field of Device 1 is 8.4 Oe, it is less than 7 Oe for Device 2.

As our structures effectively behave as single magnetic domains with well defined uniaxial magnetic anisotropy, we may determine for any given magnetic rotation due to current, the magnitude of an effective in-plane field (H_eff_) applied perpendicular to the easy axis of the ellipse that would induce the same rotation. Based on this we can see that for Devices 1 and 2, the induced H_eff_ is similar for similar angles between the current direction and magnetization (see Supplementary Fig. S6).

Oersted fields (**H**_Oe_) of opposite signs are generated by currents through the bottom Ta-layer and the top capping Ti layer. As the thickness of the Ta-layer is higher and its resistivity is lower, we expect that the sign of **H**_Oe_ to be consistent with the current flow in the Ta layer; namely, (**J **× **z**), where **J** is the current density and **z** is unit vector perpendicular to film plane. A similar term is associated with the Rashba effect at the interface between the HM and the magnetic layer ${{\bf{H}}}_{{\rm{F}}}\propto {\alpha }_{r}({\bf{J}}\times {\bf{z}})$, where *α*_*r*_ is the spin-orbit coupling constant. In our case, *α*_*r*_ is negative so the direction of **H**_Oe_ and **H**_F_ are opposite to one another. We note that this term is determined by the current and it is independent of the magnetization direction. As our results clearly indicate dependence of H_eff_ on magnetic orientation, it appears that the anti-damping effective field $({{\bf{H}}}_{{\rm{AD}}})\propto {\rm{m}}\times ({\bf{z}}\times {\bf{J}})$, where m is the magnetization vector, plays an important role. We note that H_AD_ is expected to be highest when m is perpendicular to spin polarization vector (***σ*** = **z **× **J**), i.e. *β* = 90° where *β* is the angle between **m** and ***σ***, which is qualitatively consistent with our observations (see Fig. S6 of Supplementary). Deviations in H_eff_ vs sin *β* curve from linearity are attributed to contributions of field-like terms. **H**_AD_ is perpendicular to the film plane and it induces an in-plane magnetization reorientation^29^ (see the supplementary material).

### SOT induced magnetization switching

**Figure 4:**
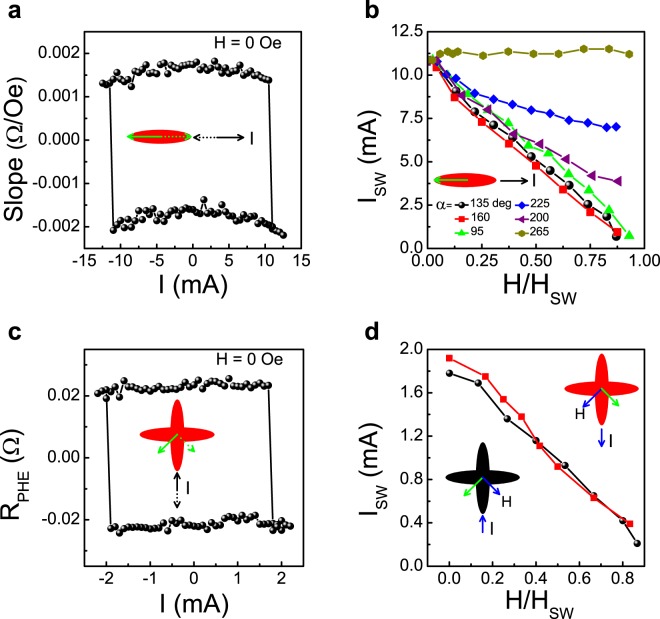
Current-induced switching in Devices 1 and 3. Device 1: (**a**) Slope of R_PHE_ vs H_⊥_ after a pulse of magnitude I is applied parallel to the long axis of the ellipse. The schematic illustrations of the initial states and the corresponding current direction are shown by arrows. (**b**) The switching current I_SW_ vs field applied at different angles normalized by the corresponding switching fields. Device 3: **(c)** Current-induced switching between the stable states by flowing a current along *y*-axis. **(d)** I_SW_ between two remanent states vs normalized field for reversed current directions.

We turn now to explore current-induced switching of our devices. Device 1 consists of a single ellipse and since the PHE is symmetric with **m**, the two remanent states give rise to the same PHE signal. Consequently, to distinguish between the states we need to perturbate the remanent state with small fields applied perpendicular to the long axis of the ellipse, as the sign of the slope of the PHE signal vs perpendicular field reflects the direction of the remanent state. Figure 4(a) shows measurements performed with zero applied field where after driving current pulses of varying magnitudes parallel to the long axis, the slope is probed. The results indicate very sharp switchings between the stable magnetic states. The observed switching current is ~10.85 mA which corresponds to current density of 5.8 × 10^7^ A/cm^2^ through Ta (see details in supplementary section), similar to the current density 3.7 × 10^7^ A/cm^2^ and 4.3 × 10^7^ A/cm^2^ through Ta reported by Liu *et al*^26^. and Fukami *et al*.^35^, respectively, to switch the magnetization of an in-plane magnetized nanomagnet. Switching with current alone is also realized in Device 3. Here, however, there are remanent states along different axes which give rise to different PHE signals. Figure 4(c) shows sharp current induced magnetization switching between the two easy axes with currents along *y*-axis below 2 mA.

As the above anti-damping like term plays an important role in Device 1, we note that the critical current density for magnetization reversal due to anti-damping torques (*τ*_*ST*_) is given by^26,29,40^

2 $${{\rm{J}}}_{{\rm{SW}}}=\frac{2e{\mu }_{0}{{\rm{M}}}_{{\rm{S}}}{{\rm{d}}}_{{\rm{FM}}}\eta \,({{\rm{H}}}_{{\rm{K}}}+{{\rm{M}}}_{{\rm{eff}}}\mathrm{/2})}{{\theta }_{{\rm{SH}}}\hslash }$$

where M_eff_ is the effective magnetization arising from the demagnetization and surface anisotropy, d_FM_ is the NiFe thickness and *θ*_SH_ is the spin-Hall angle which reflects the efficiency of generating spin current. Using *μ*_0_Ms = 1 T^29^, *μ*_0_M_eff_ = 0.7 T^41^, *η* = 0.016^42^, *μ*_0_H_K_ = 0.84 mT, we obtain *θ*_SH_ ~ 0.048, which is within the range of the reported value (0.02 to 0.22) in the literature. The current induced H_AD_ can be estimated as b/*γ* = (*ħ*J_SW_*θ*_SH_/2e*μ*_0_M_s_d_FM_) ≈ 57 Oe which is equivalent to a perpendicular field of magnitude 57 Oe applied on the sample.

To explore the dependence of the switching current (I_SW_) on the external applied field in Device 1 (Fig. 4(b)), the latter is varied between zero and the switching field in the low current limit for different values of *α*. We note that higher switching currents are required when the y component of the field is positive. Furthermore, for *α* = 265 deg, I_SW_ first increases with field slightly and then saturates up to the switching field. Figure 4(d) shows the field dependence of I_SW_ between two remanent stable states by reversed current direction in Device 3. Both of the curves display exactly the same dependence on field, indicating that the effective field induced by both directions of current overcomes the energy barrier identically. We also observed the switching by driving the current along *y*-axis in Device 1 (see Fig. S7 of supplementary material for details).

## Discussion

The results clearly indicate that in addition to torques associated with Oersted fields and field-like terms, anti-damping like torques also exist. Particularly, we can determine that the switching in Device 1 is dominated by the anti-damping like torque as both the Oersted field and the field-like effective field point toward the perpendicular direction of the long axis of the ellipse, which by itself cannot drive the switching. Magnetization reversal in configurations similar to that of Device 1 have been considered by Fan *et al*.^29^ who have shown by using second order approximation that in-plane magnetization rotation may be realized with the assistance of the anti-damping torque. In addition, Fukami *et al*.^35^ have demonstrated with macrospin simulations that a misalignment as small as one degree between the current and the in-plane magnetization may also yield such a magnetization rotation.

We note that our devices are not optimized for lowest current density and that field-free switching may be decreased significantly by decreasing the width of the Ta-arm to match the width of the ellipses. Further reduction of switching current is possible by reducing the anisotropy field of the ellipses by decreasing the NiFe thickness. Anyway, the clear demonstration of field free switching of magnetic structures with in-plane magnetization and more than one easy axis induced by shape, open exciting opportunities for practical spintronics devices, including multi-level magnetic random access memory (MRAM), where one of the magnetic layers of the MTJ is replaced with our devices or a similar device with a larger number of overlapping ellipses which enable a larger number of states per MTJ^43^, with SOTs used for write operation.

## Methods

The films are deposited on thermally oxidized Si-wafer in ion-beam sputtering chamber. The details of film deposition is described elsewhere^44^. First-stage photo-lithography followed by Ar-ion etching are performed to realize the two crossing elongated rectangles of various overlapped area. To deposit the contact pad, second-stage of photo-lithography and gold sputtering in DC-sputtering chamber have been done. Finally, e-beam lithography has been carried out to pattern an elongated ellipse of 2 × 16 *μ*m^2^ dimension on the center of the overlapped Ta-arm area and two crossing ellipses whose crossing area centered at the overlapped Ta-arm region. During the Ar-ion etching to realize the ellipse, we have over-etched into the Ta-layer to ensure the bottom of NiFe layer are fully patterned. The scanning electron microscopy images of the devices are shown in Fig. 1(a,b and c), respectively. Finally, the devices are connected with the sample puck by wire bonder. The measurements have been carried out in a home-made system equipped with Helmholtz coils and a rotating sample stage with angle resolution of 0.03°. The R_PHE_ = V_*xy*_/I has been measured in a four probe geometry, which eliminates contact resistance. All measurements are performed at room temperature.

## Electronic supplementary material

Supplementary Information
