# Supplementary material for: Magnetization switching of multi-state magnetic structures with current-induced torques
Source: Sci Rep. 2018 Oct 11;8:15160. doi: 10.1038/s41598-018-33554-0 (PMC6181950; doi:10.1038/s41598-018-33554-0)
Supplement: Supplementary file 1 — Supplementary Information [file 41598_2018_33554_MOESM1_ESM.pdf]

Supplementary - Magnetization switching of multi-state magnetic structures with current-induced torques

Shubhankar Das, Liran Avraham, Yevgeniy Telepinsky,
Vladislav Mor, Moty Schultz, and Lior Klein
*Department of Physics, Nano-magnetism Research Center,
Institute of Nanotechnology and Advanced Materials,
Bar-Ilan University, Ramat-Gan 52900, Israel*
(Dated: September 7, 2018)

Characterization of ellipses

Fig. S1 shows magnetic characterization of Devices 1 and 2. The effective uniaxial magnetic anisotropy of the two devices is demonstrated in Fig. S1(a) and (d). The figures show the planar Hall resistance ($\mathrm{R_{PHE}}$) as a function of the applied field angle (α). In addition, at each angle $\mathrm{R_{PHE}}$ is also measured with the field set to zero and the obtained remanent values of $\mathrm{R_{PHE}}$ indicate alignment of the remanent magnetization with a single easy axis.

Fig. S1(b) and (e) presents switching experiments. The devices are magnetized along the easy axis and then the magnetization is switched by applying a reversing field at different angles. The angular dependence of the switching field is qualitatively consistent with effective single magnetic domain behavior with uniaxial anisotropy commonly described with the Stoner-Wohlfarth model [1],

$$E = \mathrm{K_u} \sin^2\theta - \mathrm{M_S H} \cos(\alpha - \theta) \tag{S1}$$

where $\mathrm{M_S}$ is the saturation magnetization and $\mathrm{K_u}$ is the magnetic anisotropy constant. The solid lines are fits to [2, 3],

$$\mathrm{H_{SW}} = \frac{\mathrm{H_K}}{\left[\sin^{2/3}(\alpha - \alpha_1) + \cos^{2/3}(\alpha - \alpha_1)\right]^{3/2}} \tag{S2}$$

where α_1 represents for slight misalignment of the easy axis.

Fig. S1(c) and (f) demonstrate effective single magnetic domain behavior. We apply an external magnetic field which tilts the magnetization away from the easy axis and compared the measured magnetic direction with the expected orientation based on the Stoner-Wohlfarth model, determined numerically by finding the angle θ for which E in equation (S1) attains its minimal value [1, 2, 4].

Switching experiments are also performed with Device 3 for which the easy axes lie between the long axes of the ellipses. The angular dependence of H_{SW} shown in Fig. S2 is consistent with previous reports of Telepinsky et al.[5].

The effect of the current as a function of the angle between the current and the magnetization in Devices 1 and 2

In this part we show measurements indicating that the change in magnetic orientation induced by current decreases with increasing angle between the current and the magnetization.

Fig. S3 shows for Device 1 that when current is perpendicular to the long axis of the ellipse, the current induces only very small changes in the magnetic orientation. Measurements in Device 2 (Fig. S4), for which the remanent magnetization is at 45 deg relative to the current, show a consistent tendency. We observe that when magnetization is tilted by field towards the current direction the effect of the current increases. An opposite effect is observed when the field tilts the magnetization towards a perpendicular orientation.

For a more quantitative formulation of this observation, we compare changes in magnetic orientation of the devices due to applied magnetic field along the hard axis of the ellipse with changes induced by current which allow us to determine the effective perpendicular field (H_{eff}) induced by the current in various conditions.

Fig. S5 shows changes in R_{PHE} as a function of field perpendicular to the easy axis of the ellipse in Devices 1 and 2 and the corresponding changes in θ. Comparing these results with $\Delta\theta$ vs I (Fig. 3(c) and 3(e) of main text) we can determine the induced effective field as a function of current (see Fig. S6). We clearly see the slope of H_{eff} vs current curve gradually decreases when the initial states are shifting away from current direction for both of the devices.

We attribute this observation to the anti-damping like term (H_{AD}) which attains higher values when the magnetization direction is perpendicular to spin polarization vector ($\boldsymbol{\sigma} = \mathbf{z}\times\mathbf{J}$). The insets of Fig. S6(a) and (b) show H_{eff} as a function of sinβ (β is the angle between $\mathbf{m}$ and $\boldsymbol{\sigma}$) for both of the devices. The deviation from the linear dependence points out that a small contribution from field-like term (which include both $\mathbf{H}_F$ and Oersted field) can not be ruled out.

The magnetization dynamics governed by the SOI originated from the Rashba interaction at the interface and spin Hall effect generated by the current through HM/FM can be

expressed by the Landau-Lifshitz-Gilbert-Slonczewski equation [6, 7],

$$\frac{d\mathbf{m}}{dt} = -\gamma\mathbf{m}\times\mathbf{H_{TEF}} + \eta\mathbf{m}\times\frac{d\mathbf{m}}{dt} + \mathrm{a}(\mathbf{m}\times\boldsymbol{\sigma}) + \mathrm{b}\mathbf{m}\times(\mathbf{m}\times\boldsymbol{\sigma}) \quad \text{(S3)}$$

where γ is the gyromagnetic ratio, $\mathbf{H_{TEF}}$ is the total effective field (which includes external field, anisotropy field and $\mathbf{H}_{\mathrm{Oe}}$), η is the Gilbert damping coefficient, a and b describe the field-like term and anti-damping torque like term, respectively. By considering the anti-damping torque like term as perturbation and taking the second order approximation Fan et al.[8] has shown that the in-plane magnetization reorientation can be derived from equation (S3) as,

$$\Delta\mathrm{m}_x = \frac{-\mathrm{b}/\gamma}{2\mathrm{K_u}/\mathrm{M_S} - \mathrm{H}}\sin\alpha\Delta\mathrm{m}_z \quad \text{(S4)}$$

where $\Delta\mathrm{m}_z$ is the out-of-plane magnetization reorientation and can be derived from equation (S3) using first order approximation. It can be understood from equation (S4) that for certain conditions the anti-damping torque like term may induce an in-plane magnetization rotation.

Current-induced magnetization switching

For a given critical current of magnetization switching $\mathrm{I_{SW}}$, the current density in our devices can be calculated as

$$\mathrm{J_{SW}} = \frac{\mathrm{I_{SW}}}{\frac{\rho_{\mathrm{NiFe}}}{\rho_{\mathrm{NiFe}}+\rho_{\mathrm{Ta}}}\times\mathrm{w_{Ta}d_{Ta}} + \frac{\rho_{\mathrm{Ta}}}{\rho_{\mathrm{NiFe}}+\rho_{\mathrm{Ta}}}\times\mathrm{w_{NiFe}d_{NiFe}}} \quad \text{(S5)}$$

where ρ_{NiFe} and ρ_{Ta} are the resistivity of NiFe and Ta, respectively, $\mathrm{w_{NiFe}}$ and $\mathrm{w_{Ta}}$ are the width of the ellipse and Ta-arm, respectively, and $\mathrm{d_{NiFe}}$ and $\mathrm{d_{Ta}}$ are the thickness of the NiFe and Ta layer, respectively. Using $\mathrm{I_{SW}}$ = 10.85 mA (the current for zero-field induced switching in Device 1), ρ_{Ta} = 210.5 $\mu\Omega$-cm, ρ_{NiFe} = 50 $\mu\Omega$-cm[9], $\mathrm{w_{NiFe}}$ = 2 μm, $\mathrm{w_{Ta}}$ = 16 μm, $\mathrm{d_{NiFe}}$ = 2 nm and $\mathrm{d_{Ta}}$ = 5 nm, we obtain $\mathrm{J_{SW}} = 5.8\times10^7$ A/m^2. Considering the fact that the resistivity of thin Ti-film increases abruptly by decreasing the film thickness (ρ_{Ti} = 1963$\mu\Omega$-cm, measured in 10 nm film[10]), a very small current would flow through top Ti layer. So, we neglect the small current in Ti in the current density calculation.

Fig. S7 demonstrates switching in Device 1 by driving current parallel to y-axis. The switching with current parallel to x-axis and the way the slope is measured are described in the main text.

Fig. S8 shows the normalized field dependence of I_{SW} of Device 2 for various field directions.

---

[1] Stoner, E. C. & Wohlfarth, E. P. A mechanism of magnetic hysteresis in heterogeneous alloys. *Philos. Trans. R. Soc. London, Ser. A* **240,** 599-642 (1948).

[2] Tannous, C. & Gieraltowski, J. The StonerWohlfarth model of ferromagnetism. *Eur. J. Phys.* **29,** 475487 (2008).

[3] Mor, V. et al. Planar Hall effect sensors with shape-induced effective single domain behavior. *J. App. Phys.* **111,** 07E519 (2012).

[4] Genish, I. et al. The effects of geometry on magnetic response of elliptical PHE sensors. *J. App. Phys.* **107,** 09E716 (2010).

[5] Telepinsky, Y., Mor, V., Schultz, M. & Klein, L. Shape-induced bi-stable magnetic states in submicrometer structures of permalloy films. *J. App. Phys.* **111,** 07C715 (2012).

[6] Slonczewski, J. C. Current-driven excitation of magnetic multilayers. *J. Magn. Magn. Mater.* **159,** L1-L7 (1996).

[7] Slonczcwski, J. C. Currents and torques in metallic magnetic multilayers. *J. Magn. Magn. Mater.* **247,** 324-338 (2002).

[8] Fan, X. et al. Observation of the nonlocal spin-orbital effective field. *Nat. Commun.* **4,** 1799 (2013).

[9] Grosz, A., Sheikh, M. J. H. & Mukhopadhyay, S. C. *High Sensitivity Magnetometers, Springer Series on Smart Sensors, Measurement and Instrumentation*, (Springer, New York, 2016).

[10] Fan, X. et al. Quantifying interface and bulk contributions to spinorbit torque in magnetic bilayers. *Nat. Commun.* **5,** 3042 (2014).

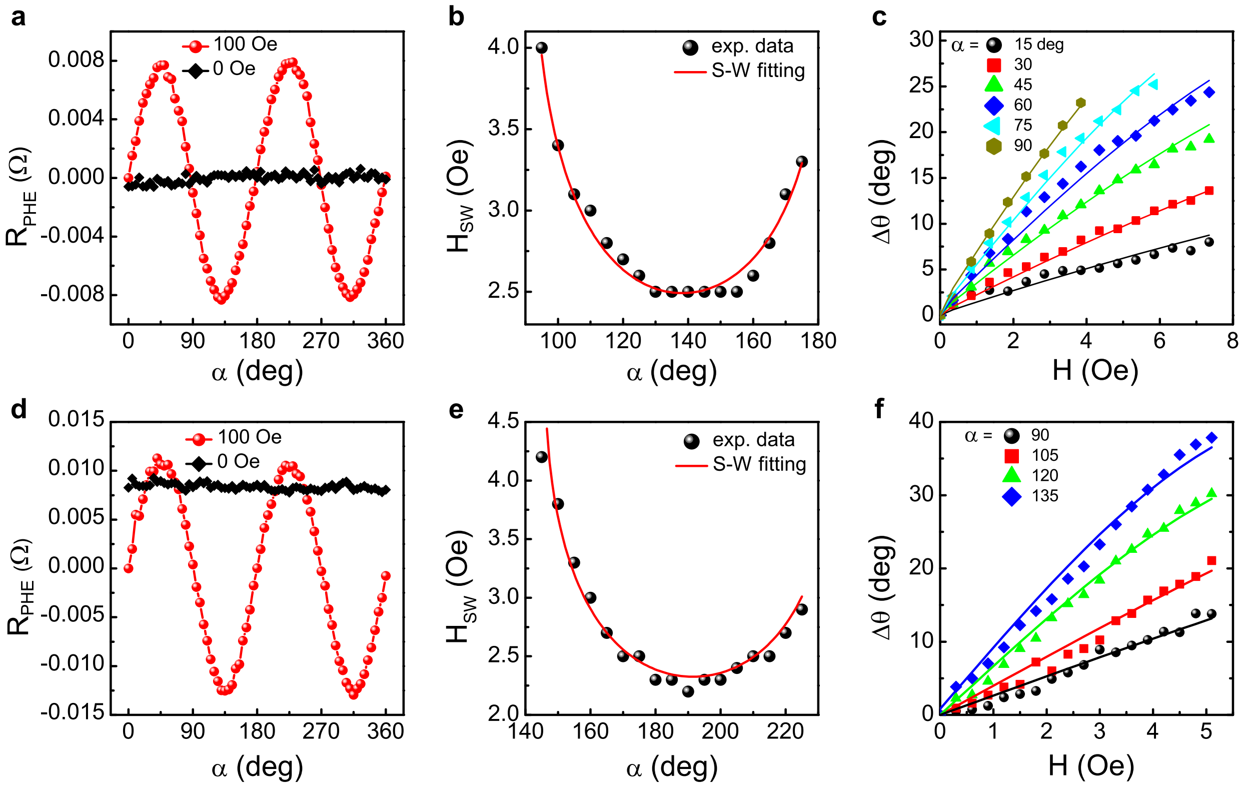

FIG. S1. **Magnetic characterization of Devices 1 and 2.** (**a**) and (**d**) R_{PHE} vs the field direction α with a field of 100 Oe and after the field is set to zero at each angle for Devices 1 and 2, respectively. (**b**) and (**e**) Switching field (H_{SW}) vs α for Devices 1 and 2, respectively. Solid lines are fits using Stoner-Wohlfart model (equation (S2)). (**c**) and (**f**) Change in magnetization orientation θ as a function of applied fields at various α for Devices 1 and 2, respectively. Solid lines are fits using Stoner-Wohlfart model (equation (S1)).

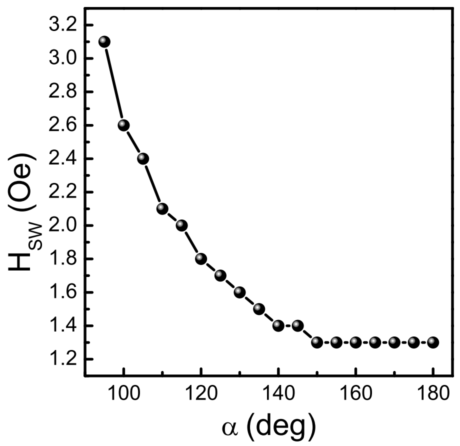

FIG. S2. **$\mathbf{H_{SW}}$ vs α for Device 3.**

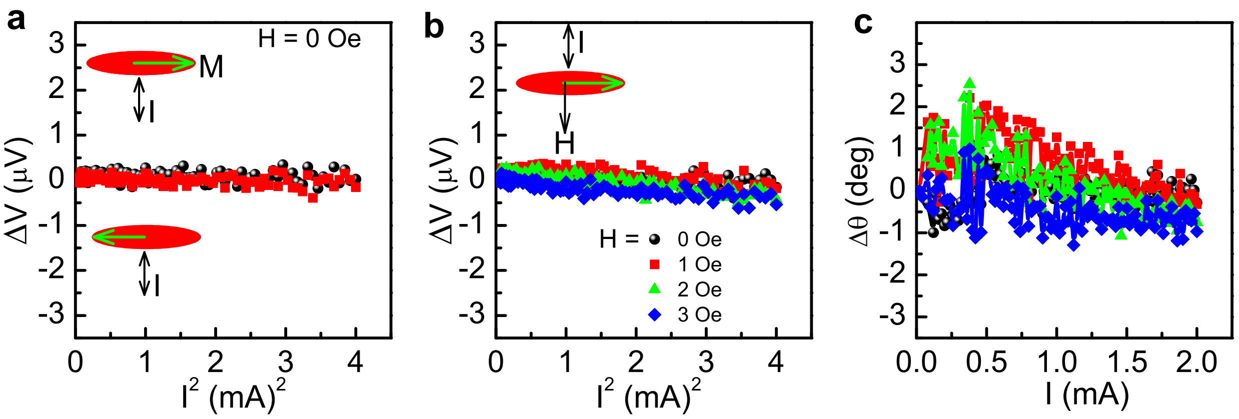

FIG. S3. **Changes in magnetic orientation in Device 1 due to current perpendicular to the long axis of the ellipse.** (**a**) Transverse ΔV vs I^2 for different remanent magnetic states. (**b**) and (**c**) ΔV vs I^2 and the corresponding change of θ, respectively, for different perpendicular magnetic fields.

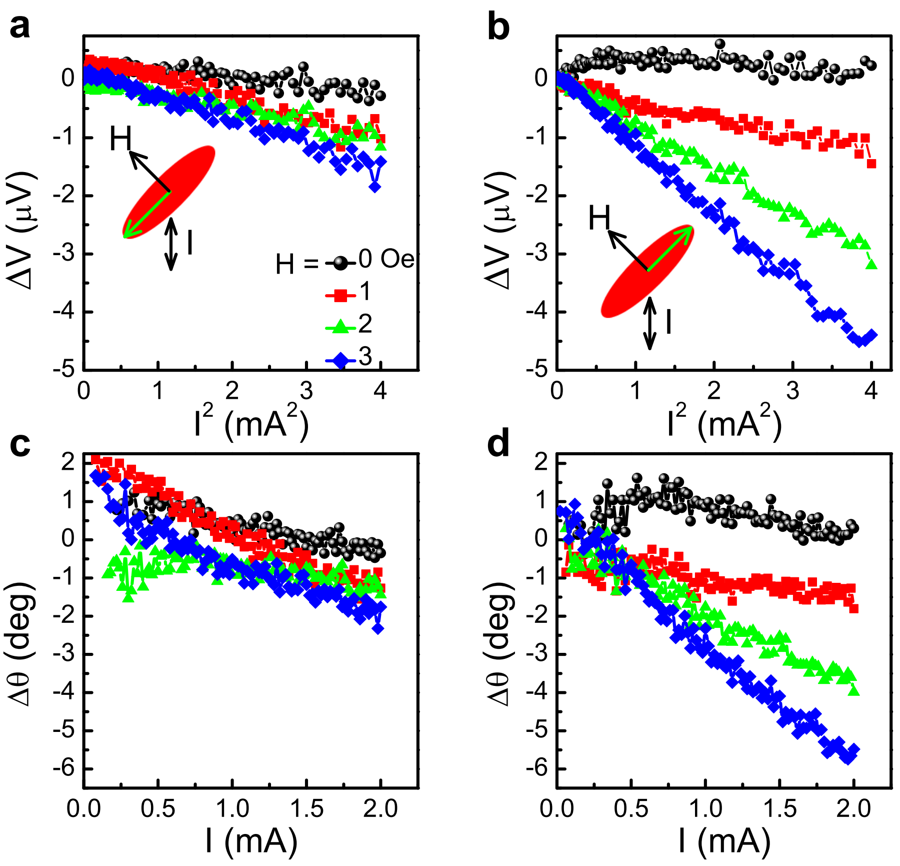

FIG. S4. **Changes in magnetic orientation due to current in Device 2.** (**a**) and (**b**) ΔV vs I^2 with different fields perpendicular to the long axis of the ellipse. (**c**) and (**d**) The corresponding change in magnetic orientation.

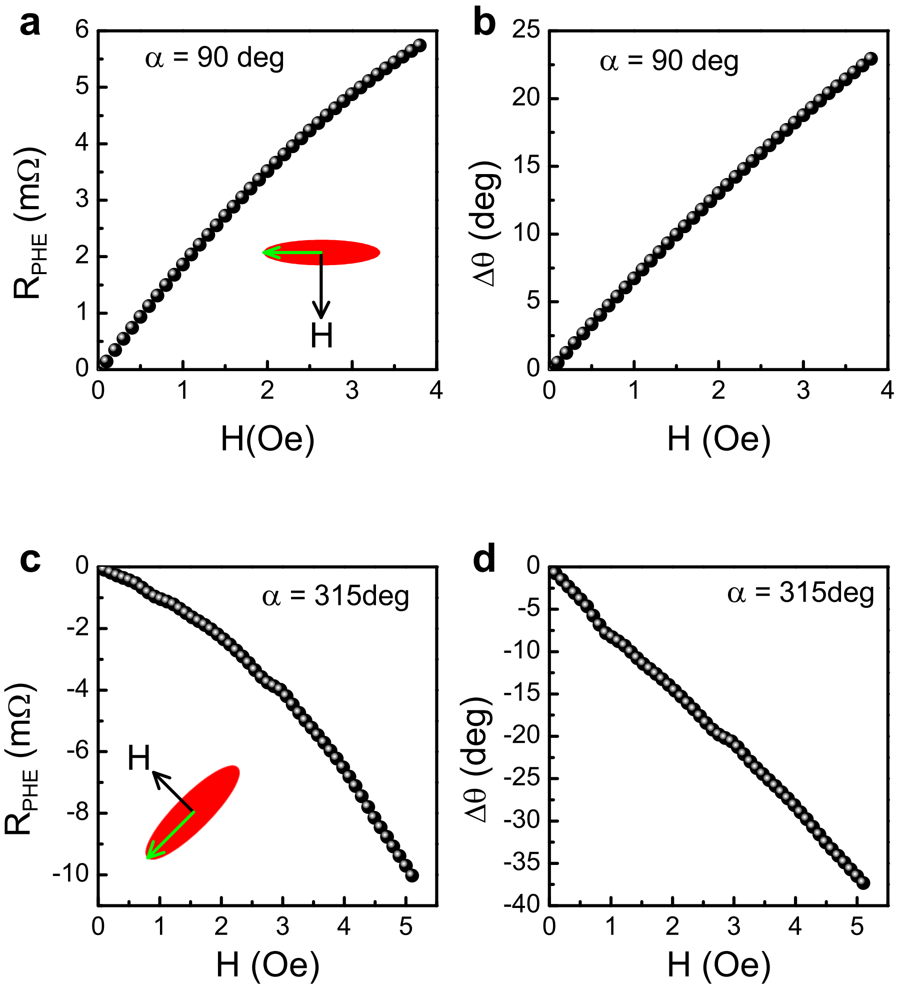

FIG. S5. **Changed in Magnetic orientation due to field applied perpendicular to the easy axis.** (**a**) and (**b**) R_{PHE} vs H and corresponding change in magnetic orientation ($\Delta\theta$), respectively, of Device 1. (**c**) and (**d**) Repeating (a) and (b) for Device 2.

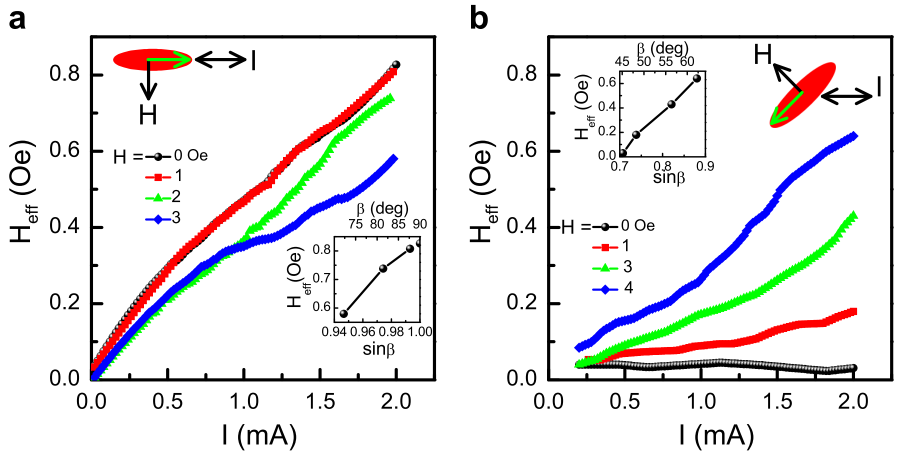

FIG. S6. **Quantification of current-induced perpendicular effective field.** (**a**) and (**b**) The H_{eff} as a function of current for different initial states for Devices 1 and 2, respectively. The current is driven along the horizontal Ta-arm. Insets show H_{eff} vs sinβ.

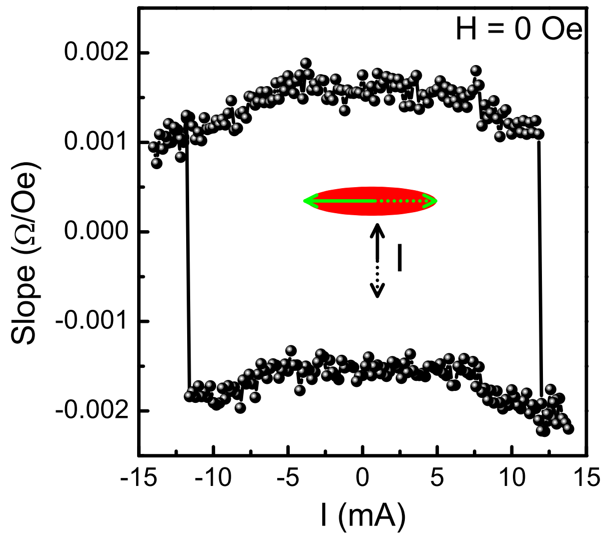

FIG. S7. **Field-free current induced switching in Device 1.** Slope of R_{PHE} vs H (see main text) as a function of current applied perpendicular to the easy axis.

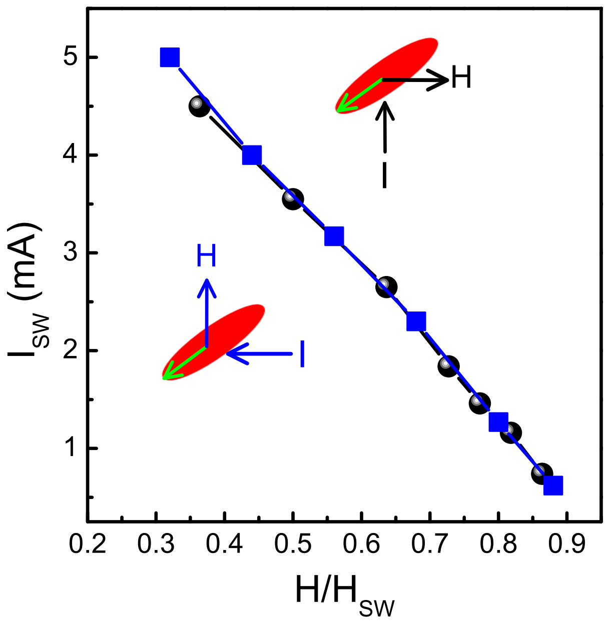

FIG. S8. **I_{SW} vs H/H_{SW} of Device 2.** Switching current as a function of normalized external field for various field directions.
